# Correction: Delayed ART initiation in “Test and Treat era” and its associated factors among adults receiving antiretroviral therapy at public health institutions in Northwest Ethiopia: A multicenter cross-sectional study

**DOI:** 10.1371/journal.pone.0304022

**Published:** 2024-05-16

**Authors:** Berihun Bantie, Gebrie Kassaw Yirga, Moges Wubneh Abate, Abreham Tsedalu Amare, Adane Birhanu Nigat, Agmasie Tigabu, Gashaw Kerebeh, Tigabu Desie Emiru, Nigusie Selomon Tibebu, Chalie Marew Tiruneh, Natnael Moges Misganaw, Dessie Temesgen, Molla Azmeraw Bizuayehu, Ahmed Nuru Muhamed, Endalk Getasew Hiruy, Amare Kassaw

The 14^th^ author’s last name is incomplete. The correct name is: Ahmed Nuru Muhamed.
