# Supplementary material for: Delayed ART initiation in “Test and Treat era” and its associated factors among adults receiving antiretroviral therapy at public health institutions in Northwest Ethiopia: A multicenter cross-sectional study
Source: PLoS One. 2022 Jul 25;17(7):e0271127. doi: 10.1371/journal.pone.0271127 (PMC9312408; doi:10.1371/journal.pone.0271127)
Supplement: Attachment — Submitted filename: PONE-D-21-39787_reviewer_v1 27 Feb.pdf [file pone.0271127.s003.pdf]

PLOS ONE

Delayed ART initiation in “Test and Treat era” and its factors among adults receiving ART at Public health institutions in Northwest Ethiopia: A multicenter cross-sectional study

--Manuscript Draft--

Manuscript Number:	PONE-D-21-39787
Article Type:	Research Article
Full Title:	Delayed ART initiation in “Test and Treat era” and its factors among adults receiving ART at Public health institutions in Northwest Ethiopia: A multicenter cross-sectional study
Short Title:	Delayed ART initiation in “Test and Treat era” and its associated factors
Corresponding Author:	Berihun Bantie Tesema, MSc<br>Debre Tabor University<br>Debre tabor, ETHIOPIA
Keywords:	Delayed ART initiation, Test and Treat era, Factors, Bahirdar , Northwest Ethiopia
Abstract:	Background : - Combined use of antiretroviral therapy (ART) has been the mainstay treatment and prevention option of HI/V AIDS. Hence, WHO has launched a universal test and treat strategy that recommends rapid or within seven initiation of ART to end HIV epidemics by 2030. However, information on timely ART initiation status in Ethiopia are scarce.<br>Method - Multicenter cross-sectional study was conducted on 400 HIV-positive adults receiving ART at public health institution of Bahirdar city. Data were extracted from patients' chart using a checklist adapted from ART intake and follow-up forms. The extracted data were entered into Epidata and exported to SPSS version 26 for statistical analysis. Both bivariable and multivariable logistic regression was executed and variables with p-values < 0.05 in the multivariable model were considered significant predictors of delayed ART initiation<br>Results- .The burden of delayed ART initiation was 156 (39%) [95% CI, 34% - 44 %). Being male (AOR 1.99, 95% 1.26 -3.16), opportunistic infections (AOR 2.5, 95%CI 1.38 4.58), chronic problems (AOR 3.7, 95%CI: 1.67 8.3), substance abuse (AOR 3.79, 95%CI: 1.9 7.4), ambulatory functional status (AOR 5.38, 95%CI 1.4- 9.6) and didn't know other HIV positive family member (AOR 1.85, 95%CI: 1.2-2.9) increases the odds of delayed ART initiation.<br>Conclusion and Recommendation- The magnitude of delayed ART initiation is found to be high. Male sex, presence of OIs and chronic problems, substance abuse, ambulatory functional status and not knowing other HIV-positive family members were identified as significant factors of late ART initiation. Special emphasis shall be considered on those with no enough social support and suffered with other comorbidities.
Order of Authors:	Berihun Bantie Tesema, MSc
	Gebrie kassaw, MSc
	Moges Wubneh Abate
	Abreham Tsedalu Amare
	Adane Birhanu Nigat, MSc
	Agmasie Tigabu, MSc
	Gashaw kerebeh, MSc
	Tigabu Desie Emiru, MSc
	Nigusie selomon Tibebu, MSc
	Chalie Marew, MSc
	Natnael Moges Misganaw, MSc

Powered by Editorial Manager® and ProduXion Manager® from Aries Systems Corporation

	Dessie Temesgen, MSc
	Molla Azmeraw Bizuayehu, MSc
	Ahmed Nuru, MSc
	Endalk Getasew Hiruy, MSc
	Amare kassaw, MSc
**Additional Information:**	
**Question**	**Response**
**Financial Disclosure**<br><br>Enter a financial disclosure statement that describes the sources of funding for the work included in this submission. Review the submission guidelines for detailed requirements. View published research articles from *PLOS ONE* for specific examples.<br><br>This statement is required for submission and **will appear in the published article** if the submission is accepted. Please make sure it is accurate.<br><br>**Unfunded studies**<br>Enter: *The author(s) received no specific funding for this work.*<br><br>**Funded studies**<br>Enter a statement with the following details:<br>• Initials of the authors who received each award<br>• Grant numbers awarded to each author<br>• The full name of each funder<br>• URL of each funder website<br>• Did the sponsors or funders play any role in the study design, data collection and analysis, decision to publish, or preparation of the manuscript?<br>• **NO** - Include this sentence at the end of your statement: *The funders had no role in study design, data collection and analysis, decision to publish, or preparation of the manuscript.*<br>• **YES** - Specify the role(s) played.<br><br>* typeset	The author(s) received no specific funding for this work.
**Competing Interests**<br><br>Use the instructions below to enter a competing interest statement for this submission. On behalf of all authors,	The authors have declared that no competing interests exist.

disclose any competing interests that could be perceived to bias this work—acknowledging all financial support and any other relevant financial or non-financial competing interests.<br><br>This statement is required for submission and **will appear in the published article** if the submission is accepted. Please make sure it is accurate and that any funding sources listed in your Funding Information later in the submission form are also declared in your Financial Disclosure statement.<br><br>View published research articles from *PLOS ONE* for specific examples.<br><br>**NO authors have competing interests**<br><br>Enter: *The authors have declared that no competing interests exist.*<br><br>**Authors with competing interests**<br><br>Enter competing interest details beginning with this statement:<br><br>*I have read the journal's policy and the authors of this manuscript have the following competing interests: [insert competing interests here]*<br><br>* typeset	
**Ethics Statement**<br><br>Enter an ethics statement for this submission. This statement is required if the study involved:<br><br>• Human participants<br>• Human specimens or tissue<br>• Vertebrate animals or cephalopods<br>• Vertebrate embryos or tissues<br>• Field research<br><br>Write "N/A" if the submission does not require an ethics statement.	Ethical clearance was obtained from the Institutional Review Board (IRB) of Bahirdar University, College of Medicine and Health Science with a protocol number of 058/2021. Then, a permission letter was obtained from the directors and ART clinic focal person of those health institutions sequentially to obtain consent to use patients' charts as a source of information.

Powered by Editorial Manager® and ProduXion Manager® from Aries Systems Corporation

General guidance is provided below. Consult the submission guidelines for detailed instructions. **Make sure that all information entered here is included in the Methods section of the manuscript.**<br><br>**Format for specific study types**<br><br>**Human Subject Research (involving human participants and/or tissue)**<br>• Give the name of the institutional review board or ethics committee that approved the study<br>• Include the approval number and/or a statement indicating approval of this research<br>• Indicate the form of consent obtained (written/oral) or the reason that consent was not obtained (e.g. the data were analyzed anonymously)<br><br>**Animal Research (involving vertebrate animals, embryos or tissues)**<br>• Provide the name of the Institutional Animal Care and Use Committee (IACUC) or other relevant ethics board that reviewed the study protocol, and indicate whether they approved this research or granted a formal waiver of ethical approval<br>• Include an approval number if one was obtained<br>• If the study involved *non-human primates*, add *additional details* about animal welfare and steps taken to ameliorate suffering<br>• If anesthesia, euthanasia, or any kind of animal sacrifice is part of the study, include briefly which substances and/or methods were applied<br><br>**Field Research**<br><br>Include the following details if this study involves the collection of plant, animal, or other materials from a natural setting:<br>• Field permit number<br>• Name of the institution or relevant body that granted permission	
**Data Availability**<br><br>Authors are required to make all data	No - some restrictions will apply

Powered by Editorial Manager® and ProduXion Manager® from Aries Systems Corporation

underlying the findings described fully available, without restriction, and from the time of publication. PLOS allows rare exceptions to address legal and ethical concerns. See the PLOS Data Policy and FAQ for detailed information.<br><br>A Data Availability Statement describing where the data can be found is required at submission. Your answers to this question constitute the Data Availability Statement and **will be published in the article**, if accepted.<br><br>**Important:** Stating 'data available on request from the author' is not sufficient. If your data are only available upon request, select 'No' for the first question and explain your exceptional situation in the text box.<br><br>Do the authors confirm that all data underlying the findings described in their manuscript are fully available without restriction?	
**Describe where the data may be found in full sentences. If you are copying our sample text, replace any instances of XXX with the appropriate details.**<br><br>• If the data are **held or will be held in a public repository**, include URLs, accession numbers or DOIs. If this information will only be available after acceptance, indicate this by ticking the box below. For example: *All XXX files are available from the XXX database (accession number(s) XXX, XXX.*).<br>• If the data are all contained **within the manuscript and/or Supporting Information files**, enter the following: *All relevant data are within the manuscript and its Supporting Information files.*<br>• If neither of these applies but you are able to provide **details of access elsewhere**, with or without limitations, please do so. For example:<br><br>*Data cannot be shared publicly because of [XXX]. Data are available from the*	All relevant data are within the manuscript.

Powered by Editorial Manager® and ProduXion Manager® from Aries Systems Corporation

XXX Institutional Data Access / Ethics Committee (contact via XXX) for researchers who meet the criteria for access to confidential data.<br><br>*The data underlying the results presented in the study are available from (include the name of the third party and contact information or URL).*<br>• This text is appropriate if the data are owned by a third party and authors do not have permission to share the data.<br><br>* typeset	
Additional data availability information:	

Powered by Editorial Manager® and ProduXion Manager® from Aries Systems Corporation

Manuscript
Click here to access/download;Manuscript;Delayed ART, PloS one.docx

Delayed ART initiation in "Test and Treat era" and its associated factors among adults receiving ART at Public health institution in Northwest Ethiopia: A multicenter cross-sectional study

Berihun Bantie Tesema [1]*, Gebrie kassaw[1], Moges Wubneh Abate [1], Abreham Tsedalu Amare[1], , Adane Birhanu Nigat[1], Agmasie Tigabu[1] , Gashaw kerebeh[2], Tigabu Desie Emiru [2], Nigusie Selomon Tibebu[2], Chalie Marew[2], Natnael Moges Misganaw [2] ,Dessie Temesgen[3], Molla Azmeraw Bizuayehu[3] , Ahmed Nuru[4] , Endalk Getasew Hiruy[5] , Amare Kassaw [2]

1. Department of Comprehensive Nursing, College of health science, Debre Tabor University Northwest Ethiopia
2. Department of Pediatrics and Child Health, College of health science, Debre Tabor University, Northwest Ethiopia
3. Department of Nursing, College of Health Science, Woldia University, Northeast Ethiopia
4. Department of Nursing, College of Medicine and Health science, Wolkite University, Southern Ethiopia
5. Department of professional nurse specialty, Saint Peters comprehensive specialized hospital, Addis Ababa, Ethiopia

*Cross ponding autor - Berihun Bantie Tesema
Po.Box - 271
Email- berihunbante@gmail.com

Email address of coauthors

GK- gebriekassaw27@gmail.com
MUA- wmoges7@gmail.com
ATA- tsedalu136@gmail.com
ABN- adanebirhanu23@gmail.com
AT- ethiomom23@gmail.com
GK- gashawkerebeh22@gmail.com
TDE – tigabud05@gmail.com
NST- nigie1221@gmail.com
CM- marewcha@gmail.com
NM- natumoges611@gmail.com
DT- Des52.8t@gmail.com
MAB- molla2ab@gmail.com
AN- ahmednuru@gmail.com
EGH- endiget2316@gmail.com
AK- amarekassaw@gmail.com

Abstract

Background: - Antiretroviral Therapy (ART) has shown promising effects on the reduction of new HIV infection as well as HIV-related morbidity and mortality. Indeed, since 2015 G.C, WHO launched a universal test and treat strategy so as to boost its effect on ending HIV epidemics by 2030 . However, information on timely ART initiation status in Ethiopia are scarce.

Method- Multicenter cross-sectional study was conducted on 400 HIV-positive adults receiving ART at public health institution of Bahirdar city. Data were extracted from patients' chart using a checklist adapted from ART intake and follow-up forms. The extracted data were entered into Epidata and exported to SPSS version 26 for statistical analysis. Both bivariable and multivariable logistic regression was executed and variables with p-values < 0.05 in the multivariable model were considered significant predictors of delayed ART initiation

Results-.The burden of delayed ART initiation was 156 (39%) [95% CI, 34% - 44 %). Being male (AOR 1.99, 95% 1.26 -3.16), opportunistic infections (AOR 2.5, 95%CI 1.38 4.58), chronic problems (AOR 3.7, 95%CI: 1.67 8.3), substance abuse (AOR 3.79, 95%CI: 1.9 7.4), ambulatory functional status (AOR 5.38, 95%CI 1.4- 9.6) and didn't know other HIV positive family member (AOR 1.85, 95%CI: 1.2-2.9) increases the odds of delayed ART initiation.

Conclusion and Recommendation- The magnitude of delayed ART initiation is found to be high. Male sex, presence of OIs and chronic problems, substance abuse, ambulatory functional status and not knowing other HIV-positive family members were identified as significant factors of late ART initiation. Special emphasis shall be considered on those with no enough social support and suffered with other comorbidities.

Keywords – Delayed ART initiation, Test and Treat era, Factors, Bahirdar , Northwest Ethiopia

1. Introduction

By the end of 2020, UNAIDS reported that nearly 37.7 million individuals were living with HIV/AIDS worldwide; of which 20.6 million reside in Eastern and South Africa regions (1). To end HIV epidemics, the combined use of antiretroviral therapy (ART) has been the mainstay treatment and prevention option since the late 1980. ART has also provided promising effects on the reduction of new HIV infection as well as HIV-related morbidity and mortality. According to the UNAIDS 2020 report, ART has contributed to 30 and 42% reduction in new HIV infection and HIV-related mortality respectively since 2010(1).

The WHO has launched the Universal Test and Treat (UTT) strategy of HIV/ AIDS since 2015 for the success of UNAIDS 2014 three 95 ambitious goals(2) . Universal test and treat strategy was define as the initiation of ART within seven days of confirmation of HIV irrespective of the WHO clinical staging as well as CD4+ levels of the patient (2, 3) . This rapid ART initiation has been found to have numerous positive effects on reducing HIV-related morbidity and mortality. The main important effects were increasing access to ART, ensuring maximal and durable viral load suppression, restoring and preserving immune function, improve quality of life, and preventing further transmission of HIV/AIDS (4-8). Despite such remarkable benefits, delayed ART initiation has continued to a major challenge for the success of ending HIV epidemics by 2030 particularly in resource limited settings (9). For instance, In Taiwan, only 68.3 % of HIV positive individuals were initiated to ART rapidly by 2016 (4). Similarly in South Africa and Zimbabwe , only 54 % and 65% of HIV positive individuals were initiated in to ART on the same day of HIV confirmation respectively(10, 11). Though there are a limited evidences in Ethiopia, finding from a study conducted at Nekemte hospital, Western Ethiopia revealed that nearly 34% of HIV positive patients were enrolled to ART beyond seven days of diagnosis(12).

Correspondingly, in Northern Ethiopia only 41.9% of adults were initiated to ART on Same day of ART diagnosis(13).

Delayed ART initiation is associated with low immunologic response, enhancing treatment failure, increases patient hospitalization, increase burden of opportunistic infections, increase costs from chronic problems, increases morbidity and mortality of patients (13-16). Peoples living with HIV/AIDS who didn't know other ART users , had TB/HIV co infection, visit traditional healers , had CD4 count > 351 cells/ mm^3, had normal BMI status were more likely to enrolled into ART lately than their respective counterparts(12, 17, 18) . Furthermore, residence, educational status, marital status , age , alcohol abuse, distance from ART center, perceived susceptibility and severity towards HIV/ AIDS, and perceived home-based care status significantly affects the time to ART initiation(19-22). To overcome this problem, solutions such as continuous professional development on HIV/AIDS management protocol, continuous updating of management guideline, scaling up ART centers, providing client-centered follow up system , formulating fixed dose combination ART drugs and providing continuous health education and promotion activities were proposed and implemented both at national level and worldwide (23, 24).

Despite such efforts a significant portions of individuals didn't has access to ART and time of ART initiation was not adequately known for those who already enrolled into ART. Hence this study will fill the gap by estimating the magnitude of delayed ART initiation with its predictor variables.

2. Methods and Materials

Study Design and Period

An institution-based cross-sectional study was conducted among 400 individuals who has enrolled into ART from November 2016 to October 2020 at public health institutions of Bahirdar City .The

city is the area of the earliest ART service center in Amhara regional state. It is located at 565 km Northwest of Addis Ababa, the capital city of Ethiopia. Currently, the city has around 11 (two hospitals and nine health center) functional ART centers. According to the report of the Amhara regional health bearou, the city bears the highest burdens of HIV/AIDS patients load in the region by which nearly 14024 individuals were ever enrolled at those institutions for ART care and services. From the above figure, a total of 2822 HIV-positive patients were newly enrolled in to ART after the implementation of the treat-all strategy.

Study Subjects

The source population for this study was all adults receiving ART after test and treat era at public health institutions in Bahirdar city. The study population was all adults newly initiated into ART from November 2016 to December 2017 at public health institutions in Bahirdar city. PLHIV who had TB/HIV coinfection at presentation, Cryptococcus meningitis infection, and individuals of with incomplete record of HIV diagnosis and initiation date were excluded from the study.

Sample size determination and Sampling technique

The minimum sample size required to conduct this study was calculated by double population proportion formula through EPINFO version 7.2. 3.1 software. Parameters such as power 80%, 95% confidence level, 25.8% proportion of outcome in unexposed group and Odds ratio of 1.87 were considered and final a total of 400 were estimated to conduct this study. Study participants were selected by computer generated simple random sampling technique after combining unique ART number of eligible patients from all ART centers in the city.

Study Variables

The main response /dependent variable for this study is the occurrence of delayed ART initiation. Whereas variables like age, sex, level of education, marital status, occupation, residence,

catchment area, have HIV positive family member , having cell phone status , base line CD4 count, baseline WHO clinical stage, baseline functional status, baseline nutritional status, presence of OIs except Tuberculosis(TB) and Cryptococcus meningitis , baseline Substance abuse status , disclosure status were categorized as independent predictor variables.

Operational Definitions

Time to ART initiation is the time interval in a day from the confirmation of HIV status till the patient is enrolled into ART. Rapid ART initiation is defined as the initiation of ART within seven days of confirmation of diagnosis irrespective of their WHO clinical staging as well as $CD4^+$ cell counts(23, 25). The functional status of the patient is categorized as “working” if daily activities of Peoples living with HIV/AIDS was not altered due to illness, “ambulatory” if the patient was not fully working but was able to do minor tasks at home, and “bedridden” when the patient remained in bed most of the time(23) . BMI status was calculated by dividing body weight by height squared. It was classified as: well-nourished(BMI: 18.5- 24.99 Kg/m^2), undernourished (BMI <18.5 kg/m_2), and overweight (BMI ≥25kg/ m^2) .Disclosure is recorded yes when the status of the individual is disclosed to at least one individual(23) . Individuals will be categorized as substance abuse when the patient scores two or more on Cut down, Annoyed, Guilty, and Eye-opener (CAGE) substance abuse screening tool (26).

Data Collection Instrument and Procedures

The data abstraction tool was adapted from the Federal Ministry of Health (FMOH) ART follow-up, the patient intake and monitoring formats. The data was collected by three BSc Nurse Professionals with the close supervision by one master of public health expert. To assure data quality, one day training was given for both data collectors and supervisors about the purpose of the study, the way to extract relevant data, and ensure the confidentiality issue of the patient

information. In addition the consistency of the checklist against the patient chart was verified by taking 10 charts and necessary modification was done accordingly.

Data processing and analysis procedures

The data was initially coded, entered into Epi data version 4.6.0.0 and simultaneously exported to SPSS version 26 software for further statistical analysis. Median, Interquartile Range (IQR) for continuous variables and proportions for categorical variables were respectively used to summarize the data. Moreover, tables and graphs were also used to present data. Both bivariable and multivariable logistic regression were fitted to identify the predictors of delayed ART initiation and those variables with p-value <0.05 in the multivariable analysis were considered as significant predictors of delayed ART initiation. Backward elimination technique was used to select variables on multivariable logistic regression model. After fitting the final model, "Hosmer and Lomeshow" goodness of fit test was used to assess the over model goodness of the regression. In addition, the discriminative power of the study was affirmed by receiver operating characteristic curve (ROC curve) and area under curve score of greater than 70 was considered as the model had good discrimination probability.

Ethical considerations

Ethical clearance was obtained from the Institutional Review Board (IRB) of Bahirdar University, College of Medicine and Health Science with a protocol number of 058/2021. Then, a permission letter was obtained from the directors and ART clinic focal person of those health institutions sequentially to obtain consent to use patients' charts as a source of information. Besides, patient identifiers (patient's MRN number) were replaced by new identification numbers during data entry period.

3. Result

Sociodemographic characteristics

A total of 400 (100%) HIV-positive adults who were enrolled in ART following Test and Treat strategy were included for final analysis. Of these, more than half of them were females (57.3%), married (51.5%), and had positive family members (59.0%). The median age of the study participants was 32 years, with an interquartile range (IQR 27-40 =13) of years. Regarding to their residence, around 229 (57.3%) of individuals reside beyond their catchment area **(Table 1**).

Table 1 . Clinical and Behavioral characteristics of HIV positive adults receiving ART at Public health institutions in Bahirdar city , Northwest Ethiopia, From November 2016 to December 2020

Variables (sample =400)	Category	ART Initiation			
		Lately initiated	Early initiated	Total frequency	%
Sex	Male	83	88	171	42.8
	Female	73	156	229	57.3
Age category	Age 15-24	22	39	61	15.2
	Age 25-34	62	114	176	44.0
	Age 35-45	57	71	128	32.0
	Age >45	15	20	35	8.8
Marital status	Married	78	128	206	51.5
	Not Married	78	116	194	48.5
Educational status	No formal education	47	70	117	29.3
	Primary education	42	57	99	24.7
	Secondary	37	69	106	26.5
	Tertiary and above	30	48	78	19.5
Occupation	Daily-laborer	24	58	82	20.5
	Farmer	14	10	24	6.0
	Merchant	26	33	59	14.8
	House-wife	26	41	67	16.8
	Employed	44	75	119	29.8
	Student	11	12	23	5.8
	Others	11	15	26	6.5
Had positive family member	Yes	48	116	164	41.0
	No	108	128	236	59.0

Catchment area Residence	Yes	148	81	229	57.3
	No	96	75	171	42.7
Having cell-phone	Yes	214	144	358	89.5
	No	30	12	42	10.5

Others: - Includes individuals who had not any job and drivers.

Clinical, Treatment-related and Behavioral Characteristics

The median CD4 and BMI status of the study participants during their enrollment to ART was 316 cell/mm3 with an interquartile range (IQR 502- 316 cell/mm3) and 20.42 with IQR (22.67- 18.59) respectively. Besides, nearly 13 % of individuals had substance abuse to at least one of the common drugs including chat, cigarette and alcohol. About one fourth (26%) of the study participants had at least one OIs. Indeed, significant number of individuals had at least one additional chronic problem that cad add additional burden on their management or overall quality of life **(Table 2).**

Table 2. Clinical and Behavioral characteristics of HIV positive adults receiving ART at Public health institutions in Bahirdar city , Northwest Ethiopia, From November 2016 to December 2020

Variables (N=400)	Category	ART Initiation			
		Lately initiated	Early initiated	Total frequency	%
Baseline BMI Status	Under weight	37	57	94	23.5
	Normal weight	109	161	270	67.5
	Over weight	10	26	36	9.0
Baseline CD4 level	greater than 500	59	74	133	33.3
	From 200-499	54	95	149	37.3
	< 200	43	75	118	29.5
Baseline WHO staging	WHO stage I&II	113	195	308	77.0
	WHO stage III&IV	43	49	92	23.0
functional status	Working	137	180	317	79.3
	Ambulatory	14	52	66	16.5
	Bedridden	5	12	17	4.3
History of OIs	Yes	54	50	104	26.0
	No	190	106	296	74.0
Presence of Chronic Problems	Yes	23	11	34	8.5
	NO	133	233	366	91.5

Substance abuse	Yes	19	35	54	13.5
	No	225	121	346	86.5

Prevalence of late ART initiation

A total of 156 (39%, 95% CI: 34%- 44 %) HIV-positive adults were categorized as having delayed ART initiation **(Fig 1**). However, ART initiation time was greatly varied among different individuals based on their socio-demographic, clinical and treatment-related conditions of the patient.

***Figure 1*. Delayed ART initiation status of HIV- positive adults receiving ART at public health institutions in Bahirdar city, Northwest Ethiopia, 2021**

Predictors of loss to follow up

During bivariable logistic regression analysis, nine variables which had p - value < 0.25 were recruited for multivariable logistic regression analysis. In the final model, the following six variables namely sex, HIV positive family member, substance abuse, chronic disease, OIs and functional status of the patients were found to be significant predictors of delayed ART initiation at 5% of the level of significance. Accordingly, the odds of delayed ART initiation is nearly two times in males than females (AOR 1.78:- 95% CI 1.13 -2.8). HIV positive individuals who didn't knew at least one HIV positive family member had nearly two or more odds of delayed ART initiation than individuals who knew at least one family member. The odds of delayed ART initiation among individuals with at least one chronic problem is almost 3.7 times **(**95% CI: 1.67 8.3**)** than individuals with no chronic problem. Substance abused HIV positive individuals were 3.79 or more times at risk to be initiated early on ART than their counterparts (3.79, 95%CI 1.9 7.4). The odds of late ART initiation among ambulatory patients is nearly 5 times (5.38, 95%CI 1.4 - 9.6) than bedridden HIV positive patients **(Table 3).**

Table 3. Bivariable and multivariable logistic regression analysis of Delayed ART initiation among adult on ART at Public health institutions in Bahirdar city, Northwest Ethiopia, from November 2016 to December 2020

Variable	*Category*	*Outcome status*		*COR [95% CI]*	*AOR [95%CI]*	*p-value*
		Late initiated	Early initiated			
Sex	Male	83	88	2.02 (1.34 3.03)*	1.99(1.26 3.16)**	0.01
	Female	73	156	1	1	
Age category	Age 15-24	21	38	0.39 (0.25 -1.71)		
	Age 25-34	63	115	0.65 (0.27- 1.53)		
	Age 35-45	61	78	0.92 (0.38- 2.2)		
	Age >45	11	13	1		
Educational status	no formal education	47	70	0.81(0.56 1.93)		
	Primary education	42	57	0.59(0.64 2.16)		
	Secondary	37	69	0.62(0.47 1.57)		
	Tertiary and above	30	48	1		
Occupation	Daily-laborer	24	58	1		
	Farmer	14	10	3.38(1.32 8.67)*		
	Merchant	26	33	1.9 (0.95 3.84)		
	House-wife	26	41	1.53 (0.77 3.03)		
	Employed	44	75	1.42(0.78 2.59)		
	Student	11	12	2.21(0.86 5.70)		
	Others	11	15	1.77(0.71 4.41)		
Marital status	Married	78	128	1		
	Not Married	78	116	0.63 (0.738 1.649)		
Reside on Catchment area	Yes	148	81	1		
	No	96	75	1.42 (0.47 1.05)		
HIV-positive family member	Yes	48	116	1	1	
	No	108	128	2. 04 (1.33 3.11)*	1.85 (1.2 2.90)**	0.01
Having Cell _phone	Yes	214	144	1	1	
	No	30	12	0.59 (0.29 1.12)	0.5 (0.2 1.11)	0.09
CD4 category	Greater than 500	59	74	1.39 (0.837 2.31)		
	From 200-499	54	95	0.99 (0.6 1.64)		

	< 200	43	75	1	1	
WHO clinical staging	Stage I and II	115	195	0.67 (0.41 1. 06)		
	Stage III and IV	41	49	1		
Functional status	Working	136	198	1.82 (0.63 5.3)	1	
	Ambulatory	14	41	1.82 (0.46 7.12)	5.38 (1.4 - 9.6)**	0.01
	Bedridden	6	5	1	0.95 (0.2- 3.67)	0.94
History of OIs	Yes	54	50	1.66 (1.05 2.6)**	2.5 (1.38 4.58)**	0.03
	No	106	190	1		
History of chronic diseases	Yes	23	11	3.67(1.73 7.75) *	3.7(1.67 8.3)**	0.01
	No	133	233	1		
Substance Abuse	Yes	19	35	3.42 (1.88 6.24) *	3.79(1.9 7.4)**	0.01
	No	225	121	1		

1-Reference category, * statistically significant at bi-variable with 5% level of significance, ** statistically significant at multivariable with 5% level of significance, CI- confidence interval,

N.B- Since we used backward likelihood ratio to select candidate variables of delayed ART initiation, we didn't display the AOR value for variables with P- value of greater than 0.1

Hosmer and Lomeshow goodness of test for multi logistic regression revealed that the model constituting such variables is good to predict the outcome variable i.e delayed ART initiation (P-value =0.639) . Indeed the discrimination power of the model was checked by receiver operating curve and the area under the curve value **(AOC value = 0.744%)** illustrated that the model is excellent to discriminate individuals with delayed ART initiation with their counterparts **(Fig 2).**

Figure 2. **ROC curve graph showing model discrimination power among adults receiving ART following Test and Treat strategy at FHCSH, Northwest Ethiopia, 2021**

4. Discussion

There is a dearth of studies that address the time of ART initiation following the implementation of the Test and Treat strategy in Ethiopia. This study has found that about 154 (39% 95%CI: 34-44%) HIV- positive adults were initiated into the ART beyond seven days. This finding is in line with recent studies conducted in Nekemete, Western Ethiopia (12), Ekurhuleni district in South Africa (27), and Zimbabwe (28). On the other hand, this finding is slightly lower than a study conducted in Taiwan(4) which might be due to a difference in socioeconomic and educational status between the two countries.

In the current study male HIV-positive individuals has higher odds of being delayed for ART initiation than their counterparts. This finding is congruent with previous studies conducted at Nekemte(12), and South Africa(10). The possible elucidation for this might be the fact that women's decision to immediately seek ART will be highly influenced by physician's counseling than males. Furthermore, if the women were found to be HIV positive during pregnancy, lactating, labor and delivery, she will be highly reinforced to begin ART at the spot or as early as possible. Furthermore, there is a claim that global and national health policy prioritizing maternal and child health services has result in unequal access to health services, including ART services.

When compared to patients without documented baseline OIs, HIV-positive people with OIs had a two-fold higher odds of delaying ART initiation time. This finding is parallel with prior ~~researches~~ studies conducted at South wollo zone and Nekemte hospital in Ethiopia (12, 29).The main justification for the above finding might be because of concerns about potential drug interactions, poor adherence as a result of pill burden, and immune reconstitution inflammatory syndrome (IRIS), not only the patient but also the health care practitioner may decide to extend ART initiation time. Nevertheless, latest evidence and guidelines recommends for the immediate initiation of ART except for patients with TB/ HIV coinfection and Cryptococcus meningitis.

The risk of delayed ART initiation is nearly four times among HIV-positive individuals with chronic problems than their counterparts. This finding is consistent with previous systematic review and meta-analysis studies conducted before the current test and treat era in Ethiopia(19). The possible justification for this might be also due to the fear of pill burden, drug-drug interactions, toxicities, and interrupting drugs while treating both chronic problems simultaneously. It implies that continuous professional development through expanding online training, short term and long-term capacity building sessions need to strengthen. Moreover, early ART initiation has a paramount role in the prevention of the incidence of chronic problems in HIV-positive patients.

The odds of delayed ART initiation among individuals with no any HIV-positive family member is higher than individuals with an HIV-positive family member. The possible elucidation for such association might be due to the fact that HIV positive individuals who did know other ART user may got strong reinforcement, and social support than being discriminated or stigmatized when compared with their counter parts. Social support from family or family encourages HIV positive patients to early enrolled on ART as well as retain on it for the rest of their life(30).

HIV positive individuals who are abused for at least one of the common substances (chat, alcohol and cigarette) had higher odds of being late on ART initiation. The possible explanation for this finding might be due to the fact that substance abuse has an overwhelmed effect on each aspects of HIV/AIDS. Substance abusers, for example, will cause cognitive impairment, which will further hamper their decision-making abilities and rigorous adherence to their ART therapy. This means that HIV positive substance abusers will face a slew of issues, including an increased risk of opportunistic infections, accelerated disease progression, and early mortality from OIs, as well

as the illness itself. The finding is consistent with literature conducted in previous era in South Wollo , and Myanmar(29, 31)

The time it takes for HIV positive people to start taking antiretroviral therapy (ART) has a substantial association with their baseline functional status. This is congruent with studies done in Gondar and South Africa(10, 22). Patients who were ambulatory, for example, had a higher chance of being delayed in receiving ART when compared with working individuals. It could be explained by the fact that HIV positive individuals with working functional status believe they are healthy enough, and that initiating antiretroviral therapy (ART) while they are relatively healthy may not be beneficial. .

5. Conclusion and recommendation

In this test-and-treat era, the magnitude of delayed ART initiation is significantly high. Males, in particular, had a high rate of delayed ART initiation compared to their counter parts. Moreover, presence of OIs and chronic problems, substance abuse, ambulatory functional status and not knowing other HIV-positive family members were identified as significant predictors of late ART initiation. This study highlighted the importance of advocating intensive counseling and education to those high risk groups in order to achieve UNAIDS/WHO targets to end HIV epidemic by 2030. Further research shall be undertaken explore the effect of other socio-economical, behavioral, institutional and contextual factors of delayed ART initiation

Abbreviations

AOR – Adjusted Odds Ratio, BMI - Body Mass Index, CD4-Cluster Differentiation cells, CI- confidence Interval, OIs - Opportunistic Infections , PLHIV - Peoples Living with Human Immune Virus, ROC- receiver operating curve, SSA - Sub-Saharan African country, TB – Tuberculosis, UNAIDS- Joint United Nations Program on HIV/AIDS , UTT- Universal Test and Treat strategy, WHO- World Health Organization.

Declarations

Consent for publication

Not applicable

Availability of data and material

All relevant data's for this study presented within the manuscript.

Competing interests

All authors have declared that they have no conflicting interests.

Authors' contributions

BBT: Synthesizing research question, formulate objective the study , design data collection, analysis, interpretation, conclusion preparing initial manuscript draft, design of study, data collection, analysis, interpretation, conclusion, preparing initial manuscript draft

GK, MU, ATA, ABN, AT, GK, TD, NST, NM, AN, DT, MY, EG , AK- Actively participated in data collection, analysis, interpretation, and conclusion as well as recommendation of the study. Indeed, they actively participate in writing up manuscript. All authors had thoroughly read and approved the manuscript.

Acknowledgment

We would like to express our deepest gratitude to Bahirdar University, College of Medicine and Health Science for letting us to conduct this research and timely availing ethical clearance. Next, we also advance our profound thanks to staffs working at ART clinic, card room and administration position in selected public health institutions of Bahirdar town facilitating conducive environment to obtain required data for this research. Last but not least, our special thanks go to data collectors and the supervisor for their willingness, commitment, and intensive works during data collection for the betterment of this research

Funding

The authors did not receive any specific fund for this project.

References

1. United Nations Joint Program on HIV/ AIDS (UNAIDS),,(https://www.unaids.org/en/resources/fact-sheet) ,2020
2. World Health Organization(WHO),, WHO guideline on when to start ART (Fast Tracking). 2015
3. Federal Ministry of Health(FMOH),, National_comprehensive_HIV care guideline ,Ethiopiea ,,231019120457 Addis Abeba , Ethiopia, 2018.
4. Huang Y-C, Sun H-Y, Chuang Y-C, Huang Y-S, Lin K-Y, Huang S-H, et al. Short-term outcomes of rapid initiation of antiretroviral therapy among HIV-positive patients: real-world experience from a single-centre retrospective cohort in Taiwan. BMJ open. 2019;9(9):e033246.
5. Ford N, Migone C, Calmy A, Kerschberger B, Kanters S, Nsanzimana S, et al. Benefits and risks of rapid initiation of antiretroviral therapy. AIDS (London, England). 2018;32(1):17.
6. Girum T, Yasin F, Wasie A, Shumbej T, Bekele F, Zeleke B. The effect of "universal test and treat" program on HIV treatment outcomes and patient survival among a cohort of adults taking antiretroviral treatment (ART) in low income settings of Gurage zone, South Ethiopia. AIDS Research and Therapy. 2020;17:1-9.
7. Labhardt ND, Ringera I, Lejone TI, Klimkait T, Muhairwe J, Amstutz A, et al. Effect of offering sameday ART vs usual health facility referral during home-based HIV testing on linkage to care and viral suppression among adults wwith HIV in Lesotho: the CASCADE randomized clinical trial. Jama. 2018;319(11):1103-12.
8. Stafford KA, Odafe SF, Lo J, Ibrahim R, Ehoche A, Niyang M, et al. Evaluation of the clinical outcomes of the Test and Treat strategy to implement Treat All in Nigeria: Results from the Nigeria MultiCenter ART Study. PloS one. 2019;14(7):e0218555.
9. Prossie Merab Ingabire, Fred Semitala, Moses R. Kamya, Damalie Nakanjako, "Delayed Antiretroviral Therapy (ART) Initiation among Hospitalized Adults in a Resource-Limited Settings: A Challenge to the Global Target of ART for 90 of HIV-Infected Individuals", AIDS Research and Treatment, vol. 2019, Article ID 1832152, 8 pages, 2019. https://doi.org/10.1155/2019/1832152.
10. Lilian RR, Rees K, McIntyre JA, Struthers HE, Peters RP. Same-day antiretroviral therapy initiation for HIV-infected adults in South Africa: Analysis of routine data. PLoS One. 2020;15(1):e0227572.
11. Rufu A, Chitimbire V, Nzou C, Timire C, Owiti P, Harries A, et al. Implementation of the 'Test and Treat'policy for newly diagnosed people living with HIV in Zimbabwe in 2017. Public health action. 2018;8(3):145-50.
12. Bayisa L, Tadesse A, Reta MM, Gebeye E. Prevalence and Factors Associated with Delayed Initiation of Antiretroviral Therapy Among People Living with HIV in Nekemte Referral Hospital, Western Ethiopia. HIV/AIDS (Auckland, NZ). 2020;12:457.
13. Moges NA, Adesina OA, Okunlola MA, Berhane Y. Same-day antiretroviral treatment (ART) initiation and associated factors among HIV positive people in Northwest Ethiopia: baseline characteristics of prospective cohort. Archives of Public Health. 2020;78(1):1-13.
14. Turi E. Cescon A, Patterson S, Davey C, Ding E, Raboud JM, Chan K, et al. Late initiation ofcombination antiretroviral therapy in Canada: a call for a national public health strategy to improveengagement in HIV care. J Int AIDS Soc. 201.
15. Gesesew HA, Ward P, Woldemichael K, Mwanri L. Late presentation for HIV care in Southwest Ethiopia in 2003–2015: prevalence, trend, outcomes and risk factors. BMC infectious diseases. 2018;18(1):1-11.

16. Poudel KC, Buchanan DR, Poudel-Tandukar K. Delays in antiretroviral therapy initiation among HIV-positive individuals: results of the positive living with HIV study. Global health action. 2016;9(1):31550.
17. Bayisa L, Tadesse A, Mulisa D, Turi E, Tolosa T. Behavioral factors associated with delayed ART initiation among people living with HIV/AIDS (PLWH) in Nekemte referral Hospital, Western Ethiopia. International Journal of Africa Nursing Sciences. 2021;14:100280.
18. Gelaw YA, Senbete GH, Adane AA, Alene KA. Determinants of late presentation to HIV/AIDS care in Southern Tigray Zone, Northern Ethiopia: an institution based case–control study. AIDS research and therapy. 2015;12(1):1-8.
19. Belay GM, Endalamaw A, Ayele AD. Late presentation of HIV positive adults and its predictors to HIV/AIDS care in Ethiopia: a systematic review and meta-analysis. BMC infectious diseases. 2019;19(1):1-8.
20. Louis C, Ivers LC, Smith Fawzi MC, Freedberg KA, Castro A. Late presentation for HIV care in central Haiti: factors limiting access to care. AIDS Care. 2007;19(4):487-91.
21. Nyika H, Mugurungi O, Shambira G, Gombe NT, Bangure D, Mungati M, et al. Factors associated with late presentation for HIV/AIDS care in Harare City, Zimbabwe, 2015. BMC Public Health. 2016;16:369-.
22. Anlay DZ, Tiruneh BT. Late ART Initiation among adult HIV patients at university of Gondar Hospital, NorthWest Ethiopia. African health sciences. 2019 Nov 4;19(3):2324-34.
23. Ingabire PM, Semitala F, Kamya MR, Nakanjako D. Delayed Antiretroviral Therapy (ART) Initiation among Hospitalized Adults in a Resource-Limited Settings: A Challenge to the Global Target of ART for 90 of HIV-Infected Individuals. AIDS Research and Treatment. 2019;2019:1832152.
24. Plazy M, Newell ML, Orne-Gliemann J, Naidu K, Dabis F, Dray-Spira R. Barriers to antiretroviral treatment initiation in rural K wa Z ulu-N atal, S outh A frica. HIV medicine. 2015;16(9):521-32.
25. Organization WH. Guidelines for managing advanced HIV disease and rapid initiation of antiretroviral therapy, July 2017. 2017.
26. Ewing JA. Detecting alcoholism. The CAGE questionnaire. Jama. 1984;252(14):1905-7.
27. Chauke P, Huma M, Madiba S. Lost to follow up rate in the first year of ART in adults initiated in a universal test and treat programme: a retrospective cohort study in Ekurhuleni District, South Africa. The Pan African medical journal. 2020;37:198-.
28. Rufu A, Chitimbire V, Nzou C, Timire C, Owiti P, Harries A, et al. Implementation of the 'Test and Treat'policy for newly diagnosed people living with HIV in Zimbabwe in 2017. Public health action. 2018;8(3):145-50.
29. Abaynew Y, Deribew A, Deribe K. Factors associated with late presentation to HIV/AIDS care in South Wollo ZoneEthiopia: a case-control study. AIDS research and therapy. 2011;8(1):1-6.
30. Mi T, Li X, Zhou G, Qiao S, Shen Z, Zhou Y. Hiv disclosure to family members and medication adherence: role of social support and self-efficacy. AIDS and Behavior. 2020;24(1):45-54.
31. Linn KZ, Shewade HD, Htet KKK, Maung TM, Hone S, Oo HN. Time to anti-retroviral therapy among people living with HIV enrolled into care in Myanmar: how prepared are we for 'test and treat'? Global health action. 2018;11(1):1520473.

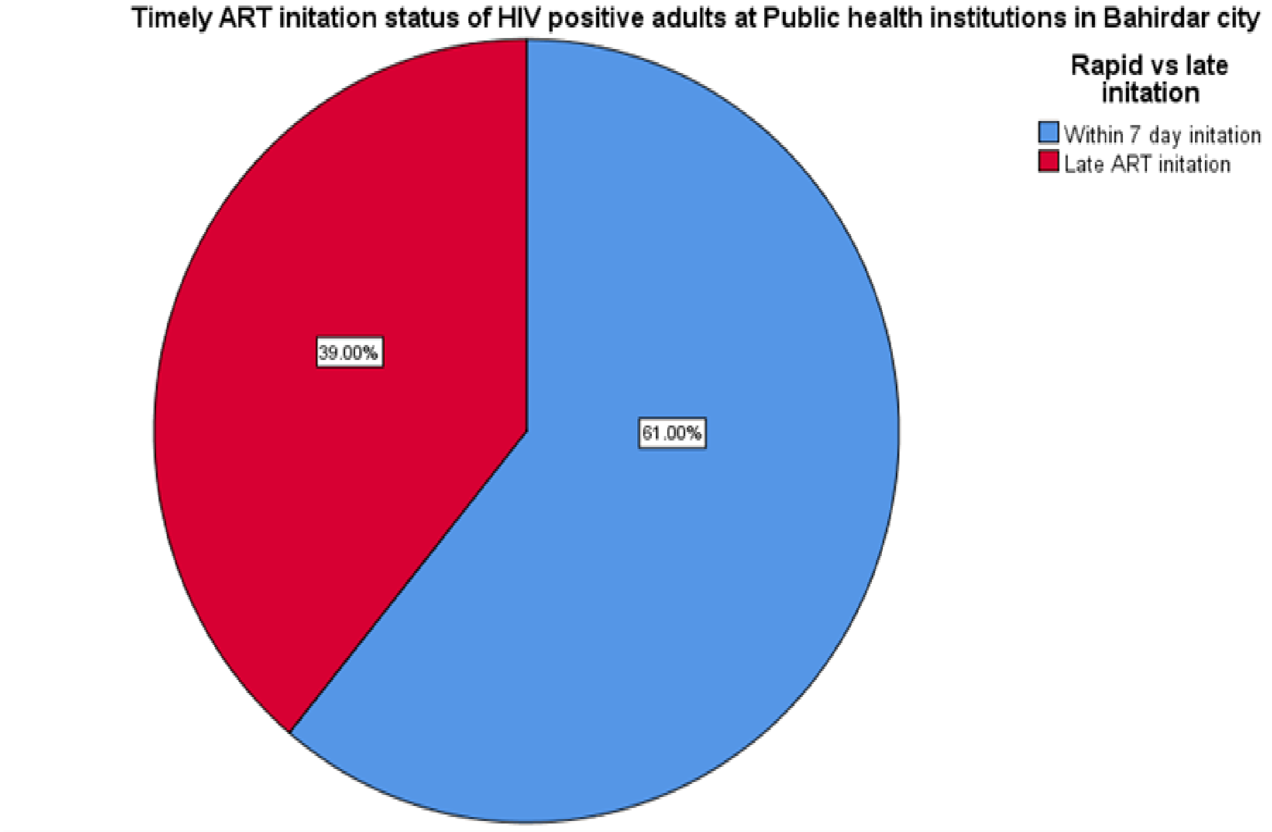

Figure 1. Delayed ART initiation status of HIV- positive adults receiving ART at public health institutions in Bahirdar city, Northwest Ethiopia, 2021

Figure

Click here to access/download;Figure;ROC analysis result of the final model.pdf

ROC analysis result of the final model in delayed ART initiation

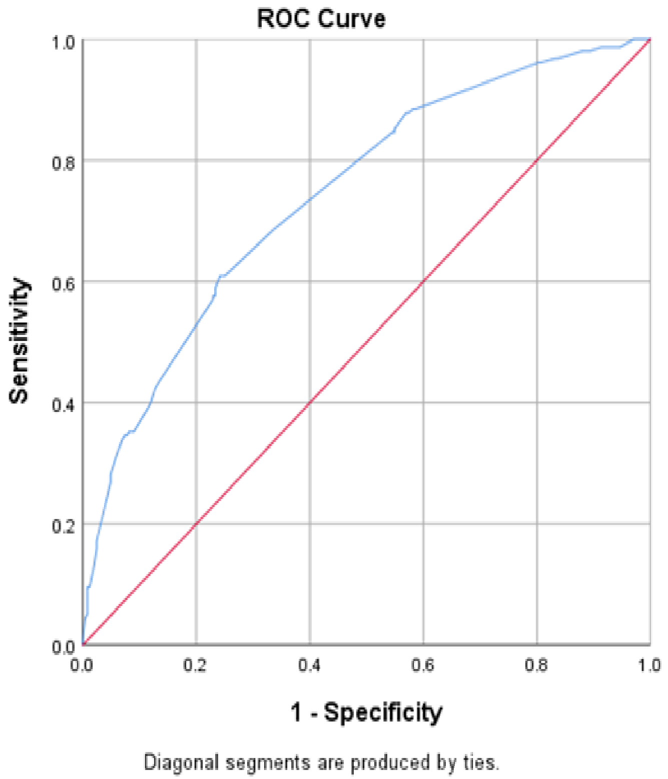

Figure 2. ROC curve graph showing model discrimination power among adults receiving ART following Test and Treat strategy at FHCSH, Northwest Ethiopia, 2021
