# Supplementary material for: Delayed ART initiation in “Test and Treat era” and its associated factors among adults receiving antiretroviral therapy at public health institutions in Northwest Ethiopia: A multicenter cross-sectional study
Source: PLoS One. 2022 Jul 25;17(7):e0271127. doi: 10.1371/journal.pone.0271127 (PMC9312408; doi:10.1371/journal.pone.0271127)
Supplement: Attachment — Submitted filename: Journal_reviewPONE-D-21-39787.docx [file pone.0271127.s002.docx]

**Manuscript Number:** PONE-D-21-39787
**Manuscript Title:** Delayed ART initiation in “Test and Treat era” and its associated factors among adults receiving ART at Public health institution in Northwest Ethiopia: A multicenter cross-sectional study

Abstract

Page 2, line 2: The background explanation is different from the first page of article.

Page 2, line7: In the patient’s chart, is HIV testing date is available? It is not clearly mentioned.

Page 2, line 14: Definition of chronic problem is not clear. Why social support is recommended?

Introduction

It would be better to include HIV morbidity and mortality data of Ethiopia.

Page 3, line 43 & Page 4, line 47: Reference 10,11 & 13, majority of patients received ART initiation within a day of diagnosis confirmed. May need to revise the sentences to show still need to reach the global target because in this study delay is defined for >7 days of diagnosis

Methods and Materials

It would be better to explain ART diagnosis and ART initiation processes. It can be performed at the same center or is there any patient take ART test from different places enrolled into ART service center? Can delay in ART initiation happen due to service factors?

**Study Design and Period**

Page 4, line 67: The author mentioned that study participants were 400 participants enrolled into ART from November 2016 to October 2020. But in page, line 78, the study population was selected from the record from November 2016 to December 2017. The study inclusion period is different.

**Study Subjects**

Page5, line 79: Why study subjects were selected from two years periods 2016-2017? Is there any changes in ART health care system after 2017? Using 2016-2017, the result may not reflect to the current situations? Adult is >15 years?

Page 5, line 80: Why TB/HIV coinfection at presentation, Cryptococcus meningitis infection are excluded from the study?

**Sample size determination and Sampling technique**

Page 5, line 85: It would be better to include the reference of using 25.8% proportion of outcome in unexposed group and Odds ratio of 1.87 for sample size calculation.

**Operational Definitions**

Page 6, line 98: It would be better to use delay initiation of ART for outcome definition.

**Result**

The name of outcome variable “Delayed ART initiation” like title should be better to use constantly in tables and texts.

Line 160, page 10: In Table 1 and Table 2, only overall frequency and percentage of all variables should be better to be mentioned without showing the classification of delay and early initiation of ART. Because number of delay ART was presented lately and presentation flow is in reverse. In addition, classified data is duplicated with Table 3 (OR) Otherwise, the information of Prevalence of late ART initiation should be better presented first in the result before the Sociodemographic characteristics of participants. What is median time to ART initiation?

In Table 1, Had positive family member or know other HIV positive family member??

In Table 2, categorization of BMI is underweight or undernourished?? It would be better to use the same. Presence of Chronic Problems or chronic health problems?? It is not clear. Need foot note for BMI, OI etc

In Table 2 and table 3, Presence of chronic problem and History of chronic diseases?? Variables names are changed. It would be better to use constant variable name through the paper.

In Table 1 and table 3, Had positive family member or HIV-positive family member or know other HIV positive family member?? It would be better to use constant variable name through the paper.

In Table 1 and table 3, Reside on Catchment area or Catchment area Residence?? It would be better to use constant variable name through the paper.

Serial of variable names in Table 3 should be the same with that in Table 1 and Table 2. In table 3, Marital status should be before education like in Table 1. The same for Reside on Catchment area. In table 3 bivariate association of BMI and delay was not included.

In Table 3, what is ** in COR and what is COR and AOR? “-“signs should be shown between numbers showing 95% CI.

Table 3, In variables of HIV-positive family member, History of OIs, History of chronic diseases and Substance, first row should be used for reference by changing row positions. It is difficult to interpret the data. It can lead to misinterpretation.

Figure 2. How ROC curved is developed and how it is useful for your study should be written in data analysis portion. It should not be 2021 in Figure 2 title. Page 12 line 189, AOC or AUC??

Page 10, line 160: Prevalence of late ART initiation: It is better to use constant outcome variable name ”Delayed ART initiation”. Prevalence?

In Figure 1, I think I should not be 2021. Name of outcome variable should be constant with that in title. If median time taken and proportion of delay in ART initiation are shown in texts, figure 1 may not need.

Page 10, line 168: What is Predictors of loss to follow up?

Discussion

May to need to discuss about generalizability of the result. Why social support is recommended for reducing delay in this study? The result can reflect to the current situation?
