# Supplementary material for: Delayed ART initiation in “Test and Treat era” and its associated factors among adults receiving antiretroviral therapy at public health institutions in Northwest Ethiopia: A multicenter cross-sectional study
Source: PLoS One. 2022 Jul 25;17(7):e0271127. doi: 10.1371/journal.pone.0271127 (PMC9312408; doi:10.1371/journal.pone.0271127)
Supplement: Attachment — Submitted filename: A rebuttal letter to commnets.docx [file pone.0271127.s004.docx]

**Dear Editor**

**Subject- To send a rebuttal letter for comments raised by editor and reviewrs**

Thank you for allowing us to submit the revised version of the manuscript entitled " *Delayed ART initiation in “Test and Treat era” and its associated factors among adults receiving ART at Public health institution in Northwest Ethiopia: A multicenter cross-sectional study* " for publication in the journal of PLOS ONE. We recognize the time and effort you and the reviewers dedicated to providing feedback on our manuscript and are grateful for the insightful comments and valuable improvements to our paper. We have incorporated the comments and suggestions given by all the reviewers and the Editor. All of the reviewers' and the editor's comments and recommendations have been taken into account. The manuscript has been extensively revised and improved as a result of the constructive remarks of the reviewers and editor. In the text, the modifications are indicated. Please find a detailed answer to the reviewers' criticisms and concerns below.

N.B- Our responses are highlighted with green color

King regards

Berihun Bantie

On behalf on the authors

***Manuscript Number***: PONE-D-21-39787

***Manuscript Title:*** Delayed ART initiation in “Test and Treat era” and its associated factors among adults receiving ART at Public health institution in Northwest Ethiopia: A multicenter cross-sectional study

**1.Comments and their responses for points raised by academic editor**

1. Please ensure that your manuscript meets PLOS-ONE’s style requirements, including for file names?

- We, the authors, carefully review the PLOS ONE formatting.pdf files and try to comply with it throughout the manuscript.

2. Please provide additional details regarding participants consent. in the ethics statement in methods and online submission forms ,please ensure you have specified what type of consent you obtained .

- As noted in the method section of manuscript, because the study was conducted retrospectively using patient’s medical record, informed consent directly from the patient was waived; instead, a supportive letter was sent from the IRB to each public health institution in Bahirar Dar City. Then a permission letter that ensures the use of patients' charts as a source of information was obtained from the directors and ART clinic focal person of those health institutions, respectively. During data collection and entry, patient identifiers (patient’s medical registration number (MRN)) were replaced by new identification numbers or codes. Besides, the collected data was treated with confidentiality.. Then the data is collected from patients chart using the permission letter. ( page 9, line# 176)

3. In your Data Availability statement, you have not specified where the minimal data set underlying the results described in your manuscript can be found?

Answer- Since the study is conducted on peoples living with HIV AIDS (PLHIV), we share the minimal data set file with no including potentially identifier of the patient ( available on additional file).

4. Please Amend either the abstract on the online submission form or the abstract in the manuscript.

- Response- We accept and made them uniform

**2. *Reviewer #1 . Abstract***

1. Please avoid abbreviation like G.C in Abstract -- **Corrected as suggested** ,Page 2, line#4-5
2. inconsistent writing pattern of 95% CI (some have hyphen and some doesn't have hyphen between the two values)- **Corrected as suggested** , Page 2,line# 16-19
3. Better to mention what is “delayed ART intimation in the abstract)-

Response- Thank you and we revise it. However, In order to maintain the flow of idea with the previous sentence , we prefer to explain about the rapid initation of ART or Universal test and treat strategy” of HIV/ AIDS by including its definition and aim so that individuals will easily comprehend the initiation of the study. --- **page 2#, Line # 3 -6**

**Introduction section**

1. Need a brief background or context of the study sites, such as when was the policy for test and treat endorsed in Ethiopia, current practice and procedures for test & treat strategy and challenges. Also about a brief on HIV epidemic of Ethiopia as well as study sites.? Answer- Accepted and **we try to incorporate such important ideas on introduction part , Page 3, Line# 30-32, 41-46,**
2. In line # 33 ,please use the correct word for “define”-

Answer- **Accepted and modified accordingly** , page3, Line# 39

3. ) line number 41, what is meant by the phrase "ART rapidly" ? same day or within 7 days?

Response**- Accepted and modified as “within seven days of confirmation of diagnosis , page 4, line # 55**

**Method and material section**

1. Line number 116, please write more about data verification: need to write how to sample these 10 charts (e.g. random picking or something else?)---

Response:-	**In order to check the consistency between the data abstraction checklist and recording system on patients chart , we use 10 randomly selected charts which are excluded for the final study , page 8,line#150**

2. Need to tell if there was missing data or not and how missing data were handled ?)-	Response- Accepted and	we just incorporate how we handle missed data. Before proceeding to descriptive and inferential analysis of the data, missed records for variables like BMI, occupational status educational status, and CD4 levels were handled and managed. Initially, we observe the missing patterns of the variables and we conclude that there is no clear missing pattern, hence we use multiple imputations missing data management method and impute those data’s accordingly. Then, sensitivity analysis was executed to check the consistency of the output of the model with original and imputed data ( Page 8, 155-158)

**Result section**

1. line number 142: what is meant by "positive" , please re-phrase as "HIV positive" to be more specific

Response- rephrased as HIV-positive, page 10, line 188

2. Table 1 and Table 2: To add foot note about % whether it is a row percentage or column percentage-

Response - Accepted and modified accordingly including %( which it indicates the column %), table 1 on page 10, table 2 on page 11

3. Line#151, writing pattern of IQR is not consistent-

Response-	Accepted and corrected accordingly, page 10 line 189, page 11 line 196

4. Line number 154: use of wording "significant number" should be rephrased. We can't say significant by seeing only the absolute numbers and percentages only-

Answer- Accepted and rephrased it as “substantial number of individuals” ( page 11, line 199)

5. line number 180 & 181 : the reference is working functional status , so, it is better to rephrase this sentence (it is written bedridden as reference)-

Answer-Accepted and reference for both of them is working functional status ( Table3, page 14)

**Figure**

- To write long form of FHCSH as figures should be stand alone and abbreviation should not be used.- Response- Accepted and modified accordingly , Figure 02, page 15

**Figures and footnote-**

- to add footnotes for every abbreviations used in figures and tables -

Response-	Accepted and foot notes are added in each figure and table , (Table 1.2.3 on page 10,11 and 13

3. ***Reviewer # 2.***

**Abstract section**

1. Page 2, line 2: The background explanation is different from the first page of article) –

Response- we accept it, and we modified it to make the two ideas consistent. Currently, the background of the abstract almost compatible with the first paragraph of the introduction. Both of them tell about the effects of ART on HIV/ AIDS and its initiation time in this test and treat era. – (*Page 2 line 3 and page 3, line 37-41)*

2. Page 2, line 7( Page 2, line7: In the patient’s chart, is HIV testing date is available? It is not clearly mentioned.) -

*Response - Yes, the patient medical record( chart) includes history sheet, progress sheet, nursing sheet and ART intake and monitoring formats where by Patients HIV testing data and result are mainly documented in history sheet and in ART intake and motoring form ( page 2,line 13) .*

3. Page 2, line 14 (Page 2, line 14: Definition of chronic problem is not clear. Why social support is recommended?)-

Answer- Thank you .It is an edition to say chronic diseases . Patient is said to has chronic diseases whenever they have a *documented history* one or more of the following diseases such as,diabetes mellitus, Hypertension (HTN), chronic heart dieses (CHD) , Stroke Chronic obstructive pulmonary diseases(COPD),asthma, chronic liver and kidney diseases and cancer in any of the body parts.

Why social support is recommended-

Response -	Thank you. The recommendation was modified as ‘ Special emphasis shall be considered for individuals with identified risk factors in order to make recommendation short and concise. However, we initially recommend social support because as it is indicated on discussion part, the likely explanation for delayed initiation for those individuals who didn’t have other HIV-positive family member and who is abuse of substance is lack of social support from the family as well as the significant others . ( page 3,line 24-25)

**Introduction section**

1. It would be better to include HIV morbidity and mortality data of Ethiopia.

Response - **Accepted and incorporated ( page 3,line 30-32)**

2. Page 3, line 43 & Page 4, line 47: Reference 10,11 & 13, majority of patients received ART initiation within a day of diagnosis confirmed. May need to revise the sentences to show still need to reach the global target because in this study delay is defined for >7 days of diagnosis **-**

**Response- Thank you. We revised accordingly to maintain its context.** (page 4, line #55-59)

**Methods and Material section**

1. It would be better to explain ART diagnosis and ART initiation processes. It can be performed at the same center or is there any patient take ART test from different places enrolled into ART service center? Can delay in ART initiation happen due to service factors?

Response -	we try incorporate about the ART diagnosis and ART initiation processes. “ **All** ART centers provide ART service starting from early diagnosis of HIV positive adults, immediately linking them to ART , monitoring their progress through continuous follow-up” page **6 , line 95-97**

**Study Design and Period**

1. Page 4, line 67: The author mentioned that study participants were 400 participants enrolled into ART from November 2016 to October 2020. But in page, line 78, the study population was selected from the record from November 2016 to December 2017. The study inclusion period is different.

Response—**The study participants were selected from November 206 to December 2020 not December 2017. This is an edition problem and we revised it accordingly**

**( page 5, line 91 and page 6,line# 105)**

- Page5, line 79: Why study subjects were selected from two years periods 2016-2017? Is there any changes in ART health care system after 2017? Using 2016-2017, the result may not reflect to the current situations?

**Response- As noted above,** **the study participants were selected from November 206 to December 2020 not December 2017. This is an edition problem and we revised it accordingly ( page 6, line 105).**

**Adult is >15 years? Response- In Ethiopian health care management system including HIV management protocol , adults were considered as those individuals whose age is ≥ 15 years of age .**

2. Page 5, line 80: Why TB/HIV coinfection at presentation, Cryptococcus meningitis infection are excluded from the study?

**Answer-** Since the latest Ethiopian or World health organization HIV management guideline recommends differing ( extending) of ART initiation time for at least 2-8 weeks for patients with TB co-infection, and 4-6 weeks for patients with Cryptococcus meningitis to prevent serious drug reactions as well as immune reconstitution inflammatory condition. Therefore, we the exclude those patients not to have a biased finding ( which results overestimation of the true finding) . (page 7 ,line 131-135) and Reference # 4 and 5

**Sample size determination and Sampling technique**

3. Page 5, line 85: It would be better to include the reference of using 25.8% proportion of outcome in unexposed group and Odds ratio of 1.87 for sample size calculation. Response -**Accepted and referenced** accordingly ( page 6,line 113)
4. **Page 6, line 98**: It would be better to use delay initiation of ART for outcome definition**.- Response- Accepted and operational definition was given for Delayed ART initiation ( page 7 ,line130)**

**Result section**

1. The name of outcome variable “Delayed ART initiation” like title should be better to use constantly in tables and texts--- **Accepted) and revised accordingly on the revised manuscript**
2. Line 160, page 10: In Table 1 and Table 2, only overall frequency and percentage of all variables should be better to be mentioned without showing the classification of delay and early initiation of ART. Because data is duplicated with Table 3. (OR) Otherwise, the information of Prevalence of late ART initiation should be better presented first in the result before the Socio-demographic characteristics of participants. What is median time to ART initiation?-

Response - Accepted and revised according to the comment, and data duplication is minimized ( page 10, line 192 , table 1 and page 11,line 202 ,,table 2)

- What is median time**- Thank you. The reason why we didn’t calculate median time is explained as follows.** Even though we categorize individuals as **Delayed** (because they didn’t initiated within seven day**s) ,** there are some individuals whose exact date of ART initiation was not still left unknown because they didn’t start taking ART through our follow up time( particularly those HIV status is confirmed ear to the completion of the study period, December 2020 ). Secondly since the Authors intention is to determine the burden of Delayed ART initiation, we did not use **Survival analysis regression models which can estimate the median time while there is censored observation**.

3. In Table 1, Had positive family member or know other HIV positive family member??- Rresponse- as individuals who” Had HIV-positive family member” is the correct one and used consistently – ( table 1, page 10,line 191)
4. In Table 2, categorization of BMI is underweight or undernourished?? It would be better to use the same. Presence of Chronic Problems or chronic health problems?? It is not clear. Need foot note for BMI, OI etc ---

Response- **Accepted and modified accordingly:_ BMI categorization is underweight , normal weight and overweight and Chronic problems is modified into chronic diseases consistently )( Tale 2, page 11 ,line 201)**

5. In Table 2 and table 3, Presence of chronic problem and History of chronic diseases?? Variables names are changed. It would be better to use constant variable name through the paper. ---

Response- **Accepted and revised accordingly** ( table 2, page 11 and table 3,page 13,line

6. In Table 1 and table 3, Had positive family member or HIV-positive family member or know other HIV positive family member?? It would be better to use constant variable name through the paper. ---

Response -As noted above Accepted and Revised accordingly ( table 1 and 3)

7. In Table 1 and table 3, Reside on Catchment area or Catchment area Residence?? It would be better to use constant variable name through the paper.

Response-	Accepted and revised accordingly ( reside on catchment area is the correct one) ( Table 1 and Table 3)

8. Serial of variable names in Table 3 should be the same with that in Table 1 and Table 2. In table 3, marital status should be before education like in Table 1. The same for Reside on Catchment area. In table 3 bivariate association of BMI and delay was not included.---- Response- The order of marital status and educational level was made to be uniform across tables ( Table 1 and Table 3 ), Bivariable anlaysis result of BMI was included in the revised manuscript which was missed unintentionally on first draft. ( Table 1,2and 3)
9. In Table 3, what is ** in COR and what is COR and AOR? “-“signs should be shown between numbers showing 95% CI. ---

Response - **Accepted and Revised Accordingly** ( table 3,page 15)

10. Table 3, In variables of HIV-positive family member, History of OIs, History of chronic diseases and Substance, first row should be used for reference by changing row positions. It is difficult to interpret the data. It can lead to misinterpretation.-

Response - **Accepted and Revised Accordingly ( Table 3, page 14 and 15)**

11. Figure 2. How ROC curved is developed and how it is useful for your study should be written in data analysis portion. It should not be 2021 in Figure 2 title. Page 12 line 189, AOC or AUC??----

**Response- T**he receiver operating characteristic curve (ROC curve), which is produced by plotting the true positive rate against the false positive rate( 1-specificity)of the model at various thresholds, was used to check the discriminative power of the model, and an area under the curve (AUC) value of greater than 70 was used to categorize the model as it can adequately classify individuals with and without the outcomes (Page 9, line 169)

12. **Page 10, line 160:** Prevalence of late ART initiation: It is better to use constant outcome variable name” Delayed ART initiation”. Prevalence**?-**

**Response- Accepted and Revised accordingly( page 12, line #205)**

13. In Figure 1, I think I should not be 2021. Name of outcome variable should be constant with that in title.

Response - The Name of the outcome variable made to be constant throughout the paper

If median time taken and proportion of delay in ART initiation are shown in texts, figure 1 may not need**. –**

**Response-** As stated above, we can’t estimate the median time to ART initiation because, the exact ART initiation time for particularly those individuals whose HIV status is known near to the completion of the study period ( December 2020) is left unknown even though we can say that they are delayed . Secondly, since graphs make the data more sound, easily interpretable, and attractive particularly for a variable with category of less than 3 or 4, we prefer to use pie chart to present our data.

14. **Page 10, line 168: What is Predictors of loss to follow up? -----**

**Response- It is to say predictors of Delayed ART initiation , so it is revised accordingly ( page 13, line #214)**

15. May to need to discuss about generalizability of the result. Why social support is recommended for reducing delay in this study? The result can reflect to the current situation**----**

Response- We incorporate **the limitations of** the study to provide a clear image of the generalizability of the study. Social support is recommended because the root cause why those high-risk individuals (those who do not have HIV-positive family members or substance abuse) end up with Delayed ART initiation will probably be a lack of strong family/social support, which motivates them to early engage in ART as well as retain them for a long time. Therefore ,we finally revised the recommendation as follows” Special emphasis shall be considered on those individuals with other comorbidities, and had not had other HIV-positive family member who will provide enough support on his/ her care”. ( page 18, line 302)

***Reviewer # 3***

**Abstract**

1. Page 2, line#7 - shouldn’t it be "public health institutions"?? Because you said multicenter cross-sectional study. Response- **Accepted and revised** ( page 1, line 10)
2. Page 2, line#10- Change bivariable in to “ simple binary logistic regression for each potential predictor would be a better usage.-

**Response- Accepted and Revised ( page 1,line #13)**

3. **Page 2, line #20- recommendation goes off evidences—**Modified to be aligned to the finding.

**Answer-** Accepted and the recommendation was modified as ‘Special emphasis shall be considered for individuals with identified risk factors” in order to make recommendation short and concise. How ever, we initially recommend social support because as it is indicated on discussion part, the likely explanation for delayed initiation for those individuals who didn’t have other HIV-positive family member and who is abuse of substance is lack of social support from the family as well as the significant others . Therefore, we indirectly recommend as social support need to be strengthen. ( page 3,line 24)

**Introduction section**

1. page 2 ,line 29- what is 30 ?– Response – It is to say 30% ( page 3,line 37)
2. page 2, line 34 -What do you mean by this? 3 goals (or) 95 goals ??

Response- this sentence is modified to make coherent and consistent with the preceding idea. However, the three 95% UNAIDS targets of 2030 states 95% of HIV-infected individuals should know their status, 95 % of individuals who knew their result should get access to (ART), and 95 % of individuals on ART should achieve virology suppression .( Page 3,line 37-39)

3. page 2,line#33,37,43, 46, 48 and 61 grammar and punctuation issues were

Response-	Thoroughly examine and edited by language experts and individuals who had good experience on writing scientific papers (page 3,line#39, page 4,,line 50,60,65,66)

4. Page 2, line 60 – at national level indicates which country—

Response – Thank you It is to indicate “Ethiopia”, so it is revised contextually (page 5,line 79-80)

**Method Section**

1. Page 5, line #79 - what do you mean by this? is it 2020 or 2017??-

Answer- **It is edition problem and revised as 2020(page 6,line 105)**

2. **Page 5,line#79,** state full words of abbreviations at initial **and** put in bracket if you use it **before -**

**Response- Accepted and revised(page 6,line 106)**

3. **Page 5, line# 85-** From which literature you took you sampling parameters -

**Response- We miss to cite it initially and we revised it now (page 6,line 113)**

4. Page 6,line# 95--- disclosure status of what? -

Response- Accepted and modified as “HIV disclosure status of HIV positive patients “(page 7,line 124)

5. Page 6, line 103 and 105- Citation for reference of BMI operational definition

Response-	reference for operational definitions were addressed (Page 7,line 136)

6. Page 6,line 105- status of what –

Response- Revised as “Disclosure is recorded as “yes” when one’s HIV positive status is informed to at least one individual ( page 8,line 139

7. **Page 7, line 117-** provide more details of selection of variables for the models and more on backward elimination technique which were stated later in the results section.--

**Answer - The authors try to incorporate more information’ on how variables were selected for the final multivariable model and how backward elimination technique** is applied to obtain significant predictors of the outcome variable( page 9, 162-167)

**Result section**

1. Page 8,140 – 400( 100%)– No need to write **% ---**

**Response- Accepted and revised ( page 10,,line #187)**

2. **Page 8, table # 1 foot note (rewrite this sentence to make grammatically correct)---**

**Response- Accepted and modified accordingly ( Page 11,line #191, table 1)**

3. Page 9, line#155- spelling error-

**Response- Accepted and revised ( page 11, line #198)**

4. Page 10, line #160= You don't need 95% CI for your descriptive summary. Only state 156 (39%). ---

Response- **Accepted and it is deleted( page 12, line 206)**

5. **Page 10, line 169- 173—**better to take these points on analysis procedure part---

**Response** - As noted above we the authors try to incorporate these and other points on variable selection portion. On the other hand, we the authors consider that ideas on line# 171-173 were highly related with the result part because they entirely talks the names and categories of the variable which are identified as statistical predictors of delayed ART initiation. (page 9,line 162-166)

6. Page 12, Figure 2. Have you spell out FHCSH**---**

**Respond- It is writing error and modified accordingly( page 16,figure 2, line #241)**

**Discussion section**

1. Page 13, Line 111 and 112-	how it is related to the previous sentence

Response-	We just revise the explanation as follows to relate it with previous one and it is a hypothesis, not referenced.

- “Giving special attention towards maternal and child health services by both global and national health policies may result in females getting stronger counseling, education, and monitoring by health care providers when compared to males, and this finally might influence women’s decision to early engage in ART” ( line 16, line#259-263)

2. Page 13, line 120 – Citation needed—

Response- **Accepted and done** accordingly ( page 17,line 271)

3. Page 13, line 226- How?? Seems disconnected from your previous sentences.

**Response- This sentence is used to indirectly indicate the root cause why patients with chronic problem would be delayed.** As noted in preceding sentence, the possible explanation of delayed ART initiation for patients with other chronic diseases might be due to unjustified fear by the patients as well as by health care providers about drug-drug interactions, high pill burdens and toxicities happening as a result of treating both chronic diseases simultaneously. This suggests that health care providers professionals' knowledge of the management of chronic diseases comorbidities of HIV/AIDS may be insufficient, and patients may not receive adequate education and counseling about it. This further implies that continuous professional development of healthcare providers through expanding on job training, short term and long-term capacity building sessions needs to be strengthened. (page 17,line 277-282)

4. Page 13, line # 230,233, 236- Grammar **issues**

**Response- Accepted and Corrected accordingly (page 18, line 283,286,288)**

5. Page 13,line # 241**-seems disconnected from your previous sentence.**

**Response- - *Accepted and we decided to delete the sentence because it states about the future consequences of delayed initiation on substance abuse patients* ( page 18,line 292)**

**Recommendation and conclusion section**

1. Include limitation of the study before generalization**-**

**Response- Accepted and we try to incorporate the main limitation of the study which has to be undertaken when we interpret and generalize the finding of the study. ( page 18, line #302)**

**N.B:- The** English language issues were thoroughly assessed and edited by language experts working as a lecturer in university and individuals who have good experience in writing scientific papers and manuscripts. This goes to both editors and reviewers.
