# Supplementary material for: Delayed ART initiation in “Test and Treat era” and its associated factors among adults receiving antiretroviral therapy at public health institutions in Northwest Ethiopia: A multicenter cross-sectional study
Source: PLoS One. 2022 Jul 25;17(7):e0271127. doi: 10.1371/journal.pone.0271127 (PMC9312408; doi:10.1371/journal.pone.0271127)
Supplement: Attachment — Submitted filename: Review2.docx [file pone.0271127.s005.docx]

**Manuscript Number PONE-D-21-39787R1**

Delayed ART initiation in “Test and Treat era” and its associated factors among adults receiving ART at Public health institution in Northwest Ethiopia:-A multicenter cross-sectional study

Thanks authors,

I agree with your response on previous my comments. Generally, the authors need to modify figure and text abbreviation, and English checking.

I only have some minor comments.

In Title, should it be public health institutions? Because you included multicenter sites of public health institutions.

I think Title should not have abbreviation such as ART.

In abstract, it would be better to have long explanation for ART, HIV, AOR,)

In abstract, some time used two decimal for AOR but some used ARO. It would be better to be the same.

In background, page 3 line 42: for Ministry of Health in Ethiopia (FMOH), is it EMOH?

In Figure 1. Pie chart. It would be better to include (n=??)

It is not clear how it is useful for ROC curve analysis. There is no any discussion and it does not mentioned it’s usefulness in the methods session.

In discussion, male were more likely to delay than female. I think the first two reasons was not clear. We could not have any evidence to support the assumption of physician’s counseling to women influencing on women decision. It would be better to include reference. And then, we could not have any information of number of pregnancy in this study. Therefore, this explanation may be irrelevant to provide the reasons behind delays of male ART seeking.
