# Supplementary material for: Delayed ART initiation in “Test and Treat era” and its associated factors among adults receiving antiretroviral therapy at public health institutions in Northwest Ethiopia: A multicenter cross-sectional study
Source: PLoS One. 2022 Jul 25;17(7):e0271127. doi: 10.1371/journal.pone.0271127 (PMC9312408; doi:10.1371/journal.pone.0271127)
Supplement: Attachment — Submitted filename: Arebuttal letter for the second revsion .1.docx [file pone.0271127.s006.docx]

**Dear Editor**

**Subject- To send a rebuttal letter for comments raised by editor and reviewers**

Thank you for allowing us to submit the revised version of the manuscript entitled " *Delayed ART initiation in “Test and Treat era” and its associated factors among adults receiving Antiretroviral therapy at Public health institutions in Northwest Ethiopia: A multicenter cross-sectional study* " for publication in the journal of PLOS ONE. We recognize the time and effort you and the reviewers dedicated to providing feedback on our manuscript and are grateful for the insightful comments and valuable improvements to our paper. We have incorporated the comments and suggestions given by all the reviewers and the Editor. All of the reviewers' and the editor's comments and recommendations have been taken into account. The manuscript has been extensively revised and improved as a result of the constructive remarks of the reviewers and editor. In the text, the modifications are indicated. Please find a detailed answer to the reviewers' criticisms and concerns below.

N.B- Our responses are highlighted with green color

King regards

Berihun Bantie

On behalf on the authors

***Manuscript Number***: PONE-D-21-39787

***Manuscript Title:*** Delayed ART initiation in “Test and Treat era” and its associated factors among adults receiving Antiretroviral therapy at Public health institutions in Northwest Ethiopia: A multicenter cross-sectional study

**1.Comments and their responses for points raised by academic editor**

1. Please ensure that your manuscript meets PLOS-ONE’s style requirements, including for file names?

- We, the authors, carefully review the PLOS ONE formatting.pdf files and try to comply with it throughout the manuscript.

**2.** Specifically, the reviewer suggests modifying your title and further improving the English language. In addition, please provide more discussion on the ROC curve analysis. Please note that PLOS ONE does not provide copyediting or proofs of accepted manuscripts. We therefore recommend that you carefully review your manuscript and correct any English errors at this time.

Response— Thank you for your genuine comment. The title is modified accordingly, **The** language issues were thoroughly assessed and edited by language experts working as a lecturer in university and individuals who have good experience in writing scientific papers and manuscripts.

**. *Reviewer 1***

Response-	We, the authors, appreciate the time and effort you dedicated to providing feedback on our manuscript and are grateful for your constructive comments, which add significant value for the betterment of our paper

The data should be provided as part of the manuscript or its supporting information, or deposited to a public repository. For example, in addition to summary statistics, the data points behind means, medians and variance measures should be available

Response— Thank you for your genuine comment. Regarding to the availability of the data, we try to access all the relevant data’s on the manuscript and its supporting file( SPSS file 1) without any restriction. Additionally, summery statics measures like mean, median were selected based on the nature of the data( categorical vs continuous), and considering the distribution ( skewed and normally distributed) for continuous variables. So that we are trying to adhere on this assumption.

***Reviewer # 2.***

Initially, we, the authors, appreciate the time and effort you dedicated to providing feedback on our manuscript and are grateful for your constructive comments, which add significant value for the betterment of our paper. We have incorporated the comments and suggestions given by you. The quality of the manuscript has been significantly changed after we made strong revision based on your recommendations. Those changes are highlighted in the manuscript and below are responses which address each questions and concerns

I only have some minor comments.

In Title, should it be public health institutions? Because , you included multicenter sites of public health institutions.

Response—Thank you for your marvelous comments, It is revised accordingly ( Title)

I think Title should not have abbreviation such as ART.

Response—Accepted and revised accordingly

In abstract, it would be better to have long explanation for ART, HIV, AOR,)

Response—Accepted and revised accordingly (page 2, line #2 , page 2,line #16) , but for HIV, we consider it as a well-known abbreviation so that writing the full term of well-known abbreviation is wasteful.

In abstract, sometime used two decimal for AOR but some used ARO. It would be better to be the same.

Response—Thank you for insightful comment, we revised it to make consistent across the manuscript( page 2, line # 16-19)

In background, page 3 line 42: for Ministry of Health in Ethiopia (FMOH), is it EMOH?

Response- Thank you and it is revised accordingly ( page 3,line #42)

In Figure 1. Piechart. It would be better to include (n=??)

Response- Accepted and revised accordingly ( page 12, Figure 1)

It is not clear how it is useful for ROC curve analysis. There is no any discussion and it does not mention its usefulness in the methods session.

Response—Thank you for your genuine comment and we revised the manuscript to make it more clear . As stated in result section, ROC curve is used to differentiate the discrimination power of the model i.e if the graph of the ROC curve is above the diagonal line ( line where area under the curve value=0.5/50%) , the model is enough to discriminated those who will have delayed ART initiation( True positives) with individuals who will have not delayed ART initiation. But if the AOR value is less than or equal to 50% , the model will not good to differentiate between Delayed ART initiation with those who have rapid ART initiation.( page 9,line 169-173)

In discussion, male were more likely to delay than female. I think the first two reasons was not clear. We could not have any evidence to support the assumption of physician’s counseling to women influencing on women decision. It would be better to include reference. And then, we could not have any information of number of pregnancy in this study. Therefore, this explanation may be irrelevant to provide the reasons behind delays of male ART seeking.

Response— Thank you for you genuine comment and we revised it accordingly .The following’s are our views regarding to the first justification of why males will have delayed ART initiation compared to females.

The first reasons is linked with the women’s decision making power not only in their home, rather in health care service is highly influenced by anybody else. Therefore, If females were tested positive, and immediately got adequate counsel to begin ART soon, they will have higher probability to engage on ART soon compared to males( page 16, line#251).

- The second justification was deleted

**N.B:- The** English language issues were thoroughly assessed and edited by language experts working as a lecturer in university and individuals who have good experience in writing scientific papers and manuscripts. This goes to both editors and reviewers.
