# Supplementary material for: Delayed ART initiation in “Test and Treat era” and its associated factors among adults receiving antiretroviral therapy at public health institutions in Northwest Ethiopia: A multicenter cross-sectional study
Source: PLoS One. 2022 Jul 25;17(7):e0271127. doi: 10.1371/journal.pone.0271127 (PMC9312408; doi:10.1371/journal.pone.0271127)
Supplement: Attachment — Submitted filename: PONE-D-21-39787R2.docx [file pone.0271127.s007.docx]

Manuscript Number:  PONE-D-21-39787R2

Title:  Delayed ART initiation in “Test and Treat era” and its associated factors among adults

receiving antiretroviral therapy at Public health institutions in Northwest Ethiopia: A

multicenter cross-sectional study

Thanks authors. I agree with your response. I have no more comments on it.
